# Clinical outcomes after treatment of multiple lesions with zotarolimus-eluting versus sirolimus-eluting coronary stents (a SORT OUT III substudy)

**DOI:** 10.1186/1471-2261-12-18

**Published:** 2012-03-19

**Authors:** Troels Thim, Michael Maeng, Jens Flensted Lassen, Anne Kaltoft, Lisette Okkels Jensen, Jan Ravkilde, Per Thayssen, Søren Galatius, Evald Høj Christiansen, Thomas Engstrøm, Morten Madsen, Leif Thuesen, Hans Henrik Tilsted

**Affiliations:** 1 Department of Cardiology, Aarhus University Hospital, Aarhus, Denmark; 2 Department of Cardiology, Odense University Hospital, Odense, Denmark; 3 Department of Cardiology, Aarhus University Hospital Aalborg, Aalborg, Denmark; 4 Department of Cardiology, Gentofte University Hospital, Copenhagen, Denmark; 5 Department of Cardiology, Rigshospitalet, Copenhagen University Hospital, Copenhagen, Denmark; 6 Department of Clinical Epidemiology, Aarhus University Hospital, Aarhus, Denmark; 7 Department of Cardiology, Aarhus University Hospital, Brendstrupgaardsvej 100, 8200 Aarhus N, Denmark

## Abstract

**Background:**

Data on clinical outcomes among patients treated with the zotarolimus-eluting Endeavor™ stent versus the sirolimus-eluting Cypher™ stent favor the sirolimus-eluting stent. However, a separate comparison of clinical outcome among patients treated for multiple lesions with these stents is lacking. We performed this comparison within the SORT OUT III trial data set.

**Methods:**

Among 2332 patients randomized in SORT OUT III, 695 were treated for multiple lesions with zotarolimus-eluting (n = 350) or sirolimus-eluting (n = 345) stents and followed for 18 months. Major adverse cardiac events (MACE); composite of cardiac death, myocardial infarction, or target vessel revascularization (TVR); was the primary endpoint.

**Results:**

Zotarolimus-eluting compared to sirolimus-eluting stent treatment was associated with increased MACE rate (13.2% vs. 2.6%; hazard ratio 5.29 with 95% confidence interval: 2.59-10.8). All secondary endpoints; all cause death, cardiac death, myocardial infarction, TVR, target lesion revascularization, in-stent restenosis, and definite stent thrombosis; were observed more frequently among zotarolimus-eluting stent treated patients. For all endpoints, hazard ratios were 1.6 to 4.6 times higher than in the overall results of the SORT OUT III trial.

**Conclusions:**

We observed better clinical outcomes among patients treated for multiple lesions with the sirolimus-eluting stent compared to those treated with the zotarolimus-eluting stent.

## Background

Percutaneous coronary intervention (PCI) with drug eluting stent (DES) implantation in single coronary artery lesions has become mainstay [1]. Gradually, PCI with DES of multiple coronary artery lesions, concomitantly, has increased, and PCI of multiple lesions in patients with multivessel disease is under evaluation as an alternative or supplement to coronary artery bypass surgery [2].

In some stent trials with comparison of zotarolimus-eluting and sirolimus-eluting stents, however, stent safety and efficacy have only been evaluated in patients with single lesions [3]. In other trials and registry studies, patients with single and multiple lesions have been analyzed together [4-6]. Trials comparing the zotarolimus-eluting Endeavor™ stent and the sirolimus-eluting Cypher™ stent generally favor the sirolimus-eluting stent [7]. However, a separate analysis of data on patients treated for multiple lesions has not been reported. Considering the current use of DES to treat multiple lesions concomitantly, such a separate analysis is relevant. In the SORT OUT III trial, we compared clinical outcome among all-comers randomized to the zotarolimus-eluting Endeavor™ stent and the sirolimus-eluting Cypher™ stent [5]. In this trial, patients with multiple lesions were included. Here, we make a separate comparison of clinical outcomes among patients treated for multiple lesions with zotarolimus-eluting and sirolimus-eluting stents in the SORT OUT III trial.

## Methods

This study was approved by the local ethics committee and complied with the Declaration of Helsinki. Patients provided written, informed consent before participation. The SORT OUT III trial was registered with ClinicalTrials.gov (NCT00660478).

In the framework of the SORT OUT organization, we undertook the SORT OUT III trial; a multi-centre, open-label, randomized, all-comer trial from January 2006 through August 2007 in five Danish high-volume PCI centers [5]. We included patients 18 years or older undergoing PCI. Patients were eligible when they needed DES stent treatment of at least one coronary lesion. There were no upper limits on the number of treated lesions, treated vessels, or lesion length. When more than one lesion required treatment, the allocated study stent should be used to treat all lesions. Exclusion criteria were: inability to provide informed consent; life expectancy of less than one year; allergy to acetylsalicylic acid, clopidogrel, ticlopidine, sirolimus, or zotarolimus; or participation in another randomised trial. Concurrent diseases or advanced age did not preclude participation.

In this substudy, we analyzed clinical outcomes among patients undergoing PCI for multiple lesions (more than one) at the index PCI. When PCI of more than one lesion was needed, non-allocated DES or bare metal stents were only implanted if the allocated study stent could not be implanted.

At the time of the index PCI, we recorded cardiovascular risk factors and comorbidity and calculated Charlson comorbidity score [8,9].

Using a telephone allocation service, we randomized patients after diagnostic coronary angiography and before PCI. With block randomization according to center, patients were randomized 1:1 to receive either the zotarolimus-eluting (Endeavor, Medtronic, Santa Rosa, CA) or the sirolimus-eluting (Cypher Select or Cypher Select+; Cordis, Johnson & Johnson, Warren, NJ) stent. Patients were stratified by gender and the presence or absence of diabetes. Patients were pre-treated with at least 75 mg acetylsalicylic acid, a 300-600 mg loading dose of clopidogrel, and 5,000 IU or 70-100 IU/kg unfractionated heparin. After PCI, dual antiplatelet regimens with lifelong acetylsalicylic acid, 75 mg daily, and clopidogrel, 75 mg daily, for one year, was recommended in accordance with Danish guidelines [10].

Clinical outcomes were assessed at 18 months. The primary endpoint was major adverse cardiac events (MACE) defined as a composite endpoint of cardiac death, myocardial infarction, and target vessel revascularization (TVR). Other endpoints were all-cause death, cardiac death, myocardial infarction, TVR, target lesion revascularization (TLR) (within the stent + 5 mm in proximal and distal directions), symptom-driven observation of in-stent restenosis (within the stent + 5 mm in proximal and distal directions), and definite stent thrombosis. These endpoints have been described previously [5].

Data on mortalilty (cardiac and non-cardiac), hospital admissions, coronary angiography, repeat PCI, and coronary bypass surgery were obtained from national Danish registries (Danish Civil Registration System, National Registry of Causes of Death, Danish National Registry of Patients, the local heart registries in the five PCI centers, and the Danish Heart Register) [11-17]. These cover the entire Danish population.

Independent study monitors, blinded to treatment assignment, reviewed all repeat coronary examinations and interventions (coronary angiography, coronary balloon angioplasty, coronary stent implantation, and coronary artery bypass surgery) and classified their cause as in-stent restenosis or stent thrombosis based on review of angiograms and patient files. An independent endpoint committee, also blinded to treatment assignment, reviewed all events and classified all myocardial infarctions and deaths.

Continuous variables with a normal distribution were analyzed using the two-sample *t*-test (Cochran *t*-test if the variances were unequal) and continuous variables with a non-normal distribution using the Mann-Whitney *U *test. Categorical variables were analyzed using the Chi-square test. Endpoints were counted in the follow-up period starting on the date of the index PCI. For each endpoint, follow up continued until occurrence of the endpoint event, death, emigration, or until 18 months after stent implantation. We estimated relative risks using Cox proportional hazards regression analysis. In the analyses, patients receiving the sirolimus-eluting stent served as reference group. Analyses were performed according to the intention-to-treat principles. We used SAS software version 9.2 (SAS Institute Inc., Cary, NC, USA) and *p *< 0.05 was considered statistically significant.

## Results

Among the 2,332 patients randomized to zotarolimus-eluting or sirolimus-eluting stents in the SORT OUT III trial, 695 received PCI for multiple (> 1) lesions at the index PCI. Of these, 350 were allocated to zotarolimus-eluting stents and 345 were allocated to sirolimus-eluting stents. Baseline patient characteristics were well-balanced between the zotarolimus-eluting and sirolimus-eluting stent groups with the exception of previous PCI which was more common among those receiving zotarolimus-eluting stents (Table 1). In total, 810 lesions were treated in the zotarolimus-eluting stent group and 787 lesions were treated in the sirolimus-eluting stent group with similar lesion and procedure data, except for a small difference in the distribution of the number of treated lesions per patient (Table 2).

**Table 1:** Baseline clinical characteristics

	Zotarolimus-eluting stent (n = 350)	Sirolimus-eluting stent (n = 345)	p
Age (years)	64.8 (10.3)	64.5 (10.4)	0.71
Men	271 (77.4%)	269 (78.0%)	0.86
Family history of coronary artery disease	154 (44.9%)	141 (41.6%)	0.38
Current smokers	94 (28.1%)	100 (30.9%)	0.44
Diabetes mellitus	44 (12.6%)	52 (15.1%)	0.34
Body mass index (kg/m^2^)	27.5 (5.6)	27.2 (4.6)	0.44
Hypertension	203 (59.0%)	178 (52.7%)	0.10
Hypercholesterolemia	243 (70.4%)	233 (68.7%)	0.63
Previous myocardial infarction	130 (37.7%)	129 (38.4%)	0.84
Previous percutaneous coronary intervention	72 (20.9%)	48 (14.2%)	0.02
Previous coronary artery bypass surgery	16 (4.6%)	18 (5.3%)	0.68
Charlson comorbidity score			0.35
0	232 (66.3%)	211 (61.2%)	
1 or 2	98 (28.0%)	109 (31.6%)	
3 or more	20 (5.7%)	25 (7.2%)	
Indication for intervention			0.19
Stable angina pectoris	195 (55.7%)	177 (51.3%)	
ST-segment elevation myocardial infarction	13 (3.7%)	16 (4.6%)	
Unstable angina pectoris or non-ST-segment elevation myocardial infarction	129 (36.9%)	146 (42.3%)	
Other	13 (3.7%)	6 (1.7%)	

Categorical variables are presented as counts (frequency in percent) and continuous variables as means (standard deviation)

**Table 2:** Lesion and procedure data

	Zotarolimus-eluting stent n = 810	Sirolimus-eluting stent n = 787	p
Number of treated lesions per patient			0.04
2	230 (65.7%)	254 (73.6%)	
3	91 (26.0%)	63 (18.3%)	
> 3	29 (8.3%)	28 (8.1%)	
Number of treated vessels per patient			0.21
1	115 (32.9%)	107 (31.0%)	
2	188 (53.7%)	207 (60.0%)	
3	47 (13.4%)	31(9.0%)	
Target lesion coronary artery			0.45
Left main	17 (2.1%)	13 (1.7%)	
Left anterior descending	308 (38.0%)	267 (33.9%)	
Left circumflex	222 (27.4%)	229 (29.1%)	
Right	261 (32.2%)	276 (35.1%)	
Bypass graft	2 (0.2%)	2 (0.3%)	
Lesion type			0.09
A	156 (19.3%)	136 (17.5)	
B	336 (41.6%)	365 (47.1)	
C	316 (39.1%)	274 (35.4)	
Length of stented segment per lesion (mm)	19.5 (11.4)	19.7 (12.2)	0.77
Length of stented segment per patient (mm)	45.2 (20.8)	44.9 (20.9)	0.87
Maximal stent diameter (mm)	3.2 (0.5)	3.2 (0.5)	0.84
Use of glycoprotein IIb/IIIa inhibitors	75 (21.4%)	87 (25.2%)	0.24
Assigned study stent could not be implanted	1 (0.1%)	11 (1.4%)	0.003

Categorical variables are presented as counts (frequency in percent) and continuous variables as means (standard deviation)

The assigned study stent could not be implanted in 1 lesion in a patient allocated to the zotarolimus-eluting stent and in 11 lesions in patients allocated to the sirolimus-eluting stent. This was a statistically significant difference in stent deliverability (Table 2).

**Figure 1:**
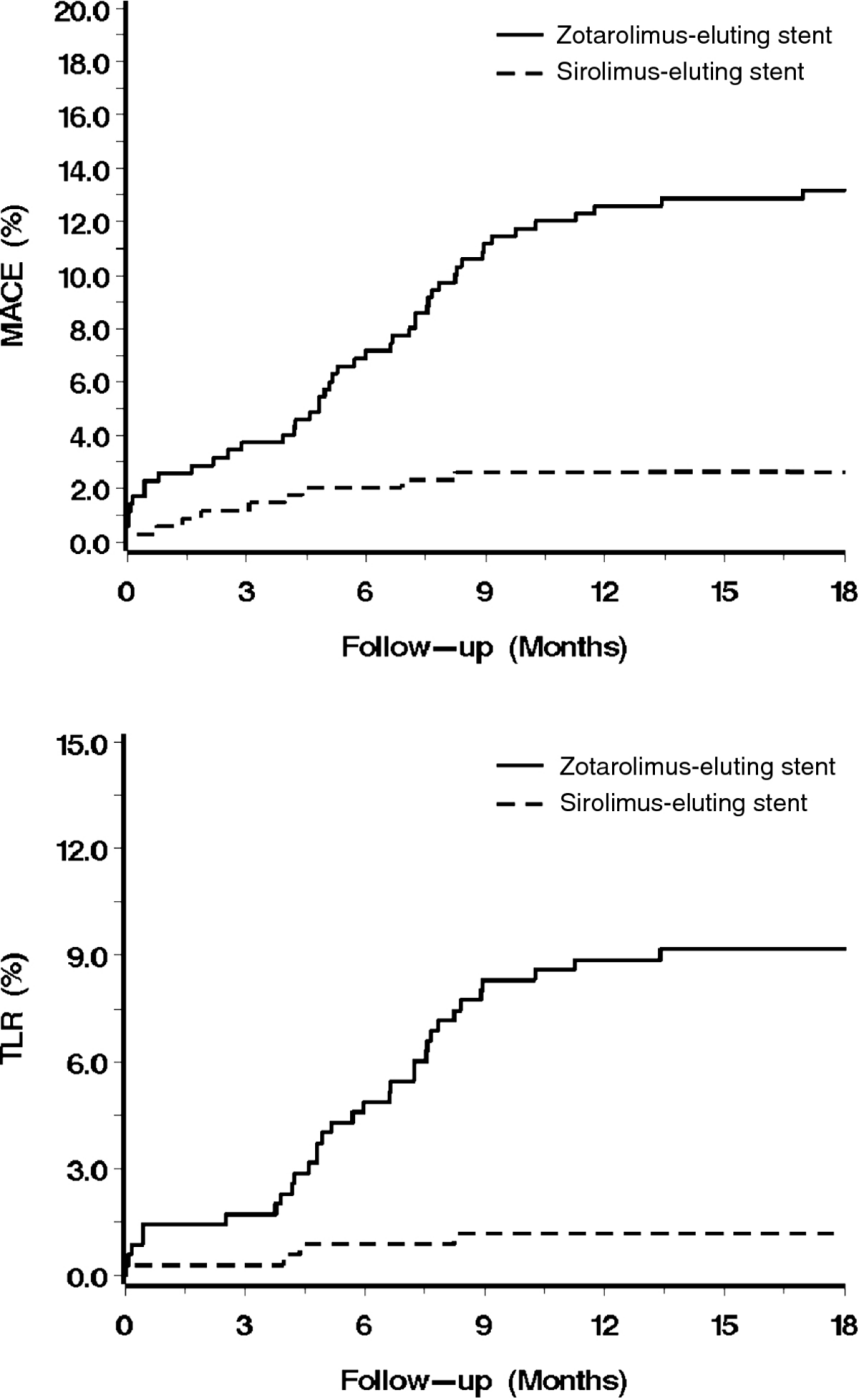
**Major adverse cardiac events (MACE) and target lesion revascularization (TLR) Kaplan-Meier curves**. MACE encompassed cardiac death, myocardial infarction, and target vessel revascularization.

All endpoints occurred more frequently in patients treated with zotarolimus-eluting stents (Table 3, Figure 1). For MACE, all cause death, myocardial infarction, TVR, TLR, and in-stent restenosis, the differences were statistically significant. For cardiac death and definite stent thrombosis, the differences were not statistically significant but the hazard ratios were high and in favor of the sirolimus-eluting stent (6.97 and 4.01, respectively).

**Table 3:** Clinical endpoints

	Zotarolimus-eluting stent (n = 350)	Sirolimus-eluting stent (n = 345)	Hazard ratio (95% confidence interval)
Major Adverse Cardiac Events	46 (13.2%)	9 (2.6%)	5.29 (2.59-10.8)
All cause death	16 (4.6%)	5 (1.5%)	3.20 (1.17-8.72)
Cardiac death	7 (2.0%)	1 (0.3%)	6.97 (0.86-56.6)
Myocardial infarction	12 (3.4%)	1 (0.3%)	12.1 (1.57-92.8)
Target vessel revascularization	37 (10.6%)	8 (2.3%)	4.79 (2.23-10.3)
Target lesion revascularization	32 (9.2%)	4 (1.2%)	8.31 (2.94-23.5)
In-stent restenosis	24 (6.9%)	3 (0.9%)	8.23 (2.48-27.3)
Definite stent thrombosis	8 (2.3%)	2 (0.6%)	4.01 (0.85-18.9)

Categorical variables are presented as counts (frequency in percent). Major adverse cardiac events; composite of cardiac death, myocardial infarction, and target vessel revascularization

## Discussion

In the present SORT OUT III sub-study, we observed better clinical outcomes among patients treated for multiple lesions with the sirolimus-eluting stent compared to those treated with the zotarolimus-eluting stent. The difference was consistent across all endpoints. Thereby, this study extends findings from previous studies to patients in need of intervention for multiple lesions [7].

Comparing the difference in clinical outcomes in the current analysis with the overall SORT OUT III study results [5], higher hazard ratios were observed for all endpoints. The hazard ratio for MACE was more than two times higher than in the overall results. The hazard ratios for all other endpoints were 1.6 (in-stent restenosis) to 4.6 (cardiac death) times higher than in the overall results. In accordance with the overall results, the outcome differences were statistically significant for MACE, all cause death, myocardial infarction, TVR, TLR, and in-stent restenosis while the differences were not statistically significant for cardiac death and definite stent thrombosis.

The observed larger difference in outcome in patients treated for multiple lesions compared to the overall results supports the relatively consistent findings in favor of the sirolimus-eluting stent [18]. Our findings are also in agreement with a subgroup analysis of the SPIRIT III trial. In SPIRIT III, the outcome among patients treated with the everolimus-eluting stent (XIENCE V) and the paclitaxel-eluting stent (TAXUS) were compared favoring the everolimus eluting stent [19]. In a subgroup analysis of patients treated for 2-vessel disease in SPIRIT III, patients treated for 2-vessel disease had a larger outcome difference in favor of the everolimus-eluting stent compared to those treated for 1-vessel disease [20].

With a true outcome difference in patients with single lesions, a similar or even more favorable outcome difference in patients with multiple lesions in general then seems likely. However, there may be effects that could not have been predicted from studies on patients with single lesions and therefore subgroup analyses or specifically designed studies should be performed in patients with multiple lesions. Also, any trial can be associated with a type 1 error, and confirmation of a trial result in similar or associated settings is always settling.

The different event rates observed could be caused by differences in drugs, drug release kinetics, polymers, or other factors related to stent design. Any of these parameters could affect plaque or vessel wall healing and thus have impact on the results. This study, however, was not designed to assess these mechanisms. Likewise, differences in stent deliverability may be attributed to many factors in stent design.

There are some limitations to our study. The SORT OUT III parent study was powered to assess the composite clinical endpoint, MACE, at 9-month follow-up [5]. Therefore, this sub-study relying on the SORT OUT III data after 18 months of follow-up was not necessarily powered to assess the examined endpoints. As well, the results obtained in this study for the rapid-release Endeavor™ stent cannot be extrapolated to other zotarolimus-eluting stents such as the slow-release Resolute™ stent [21]. The SORT OUT III trial [5], like the SORT OUT II trial [22], relied on registry-based event detection without study-related angiographic or clinical follow-up. Patient follow-up care was in accordance with normal clinical practice, i.e., a standard clinical outpatient visit at the referring hospital after 1-3 months. This patient-driven event detection reflects event presentation in routine clinical practice where patients need to contact the heath care system. This methodology thus differs from event detection based on study-driven telephone calls or visits at outpatient clinics.

## Conclusions

We observed better clinical outcomes among patients treated for multiple lesions with the sirolimus-eluting stent compared to those treated with the zotarolimus-eluting stent.

## Abbreviations

PCI: percutaneous coronary intervention; DES: drug eluting stent(s); MACE: major adverse cardiac events; TVR: target vessel revascularization; TLR: target lesion revascularization.

## Competing interests

MM has received lecture fees from Cordis, and consultant fees from Medtronic. JFL has received consultant fees from Boston Scientific, Guidant, Abbott, Terumo, St Jude medical and Cordis, and has received unrestricted research grant support from Abbott, Boston Scientific, Cordis, Medtronic, St Jude medical and Terumo. AK has received lecture fees from Cordis. LOJ has received lectures fees from Abbott and Cordis. JR has received consultant fees from Abbott and Cordis, and has received unrestricted research grant support from Abbott, Boston Scientific, Cordis, Medtronic and Terumo. LT has received lecture fees from Medtronic, Abbott, Cordis, and Boston Scientific; he also received consultant fees from Abbott and he is on the advisory board of Boston Scientific, and has received unrestricted research grant support from Medtronic, Abbott, Cordis, and Boston Scientific. All other authors declared no conflicts of interest.

## Authors' contributions

TT: analysis, interpretation of data, drafting the manuscript. MM: conception and design, acquisition of data, analysis, interpretation of data, revision of manuscript. JFL: conception and design, acquisition of data, analysis, interpretation of data, revision of manuscript. AK: conception and design, acquisition of data, analysis, interpretation of data, revision of manuscript. LOJ: conception and design, acquisition of data, analysis, interpretation of data, revision of manuscript. JR: conception and design, acquisition of data, analysis, interpretation of data, revision of manuscript. PT: conception and design, acquisition of data, analysis, interpretation of data, revision of manuscript. SG: conception and design, acquisition of data, analysis, interpretation of data, revision of manuscript. EHC: conception and design, acquisition of data, analysis, interpretation of data, revision of manuscript. TE: conception and design, acquisition of data, analysis, interpretation of data, revision of manuscript. MM: conception and design, analysis, interpretation of data, revision of manuscript. LT: conception and design, acquisition of data, analysis, interpretation of data, revision of manuscript. HHT: conception and design, acquisition of data, analysis, interpretation of data, revision of manuscript. All authors read and approved the final manuscript.

## Pre-publication history

The pre-publication history for this paper can be accessed here:

http://www.biomedcentral.com/1471-2261/12/18/prepub
